# Progress in Genito-Urinary Surgery

**Published:** 1903-06-06

**Authors:** 


(*To be continued.*)

### Progress in Genito-Urinary Surgery.

**The Diagnosis of Renal Affections.**—Improved methods of diagnosing the pathological conditions and the functioning powers of the two kidneys, separately and conjointly, have much aided the advancement of renal surgery. Some of these methods have recently been reviewed in the *Scottish Medical and Surgical Journal*.[1] Two new methods of examining for signs of disease of the kidney or ureters in cases of pyuria have been described by Bazy. One of these signs is called the *pyelo-vesical reflex*. It is elicited by pressing on the pelvis of the kidney when the patient is lying upon his back. The pressure is applied just below the margin of the ribs, and about one inch from the middle line. In certain cases this pressure produces pain which radiates down the ureter towards the iliac fossa, the groin or the testicle, and at the same time causes a desire to micturate. This sign may be obtained when there is no ordinary renal tenderness. On the other hand, if the kidney is small or the abdominal wall thick, or the liver large or tender, it may be impossible to elicit the sign. The other sign of Bazy is the so-called *uretero-vesical reflex*, and is elicited by pressing on the point where the ureter opens into the bladder. It is more practicable in women than in men. In the former the finger is introduced into the lateral pouch of the vagina, the right finger being used for the right side and the left for the left side. Pressure against the upper wall of the vagina and slight lateral movements are then made. The ureter can often then be felt rolling under the finger. The pressure excites pain and a desire to micturate if the ureter is inflamed. If cystitis only is present the ureteral orifices will not be tender, but pressure on the bladder-neck through the vagina will produce pain and a desire to micturate. Thus a diagnosis can often be effected as to whether the pus in the urine comes from the bladder or from one or both ureters. In the male enlargement of the ureters cannot be recognised owing to the presence of the vesiculæ seminales, but the same pain and desire to micturate are produced as in women when pressure is made near the ureteric orifices. Cryoscopy of the blood, that is the determination of its freezing-point, gives valuable information as to the purity of that fluid, and indirectly as to the perfection with which the renal functions are being performed. The specific gravity of a fluid indicates the weight of the solids dissolved in it; the freezing-point reveals the molecular concentration, or "osmotic pressure" as it is also called. These are not one and the same thing. Specific gravity is a *mass* effect; but the freezing-point depression is a *molecular* effect and depends on the nature of the substances dissolved. Practically a depression below normal of the freezing-point of blood, that is below from $-0{\cdot}55°$ C. to $-0{\cdot}57°$C., is usually an indication of deficient excretory power in the kidneys. The freezing-point of blood is, however, sometimes depressed in anæmia and where there is respiratory insufficiency. Also it has been found that when large abdominal tumours are present the freezing-point is sometimes depressed without renal insufficiency being the cause. Kümmell has ascertained the freezing-point of blood in 265 cases. In considering the question of nephrectomy, if the freezing-point is $-0{\cdot}56°$ C. there need be no hesitation. If it is $-0{\cdot}58°$ C., nephrectomy may be performed without permanently bad results, but the patient will probably be seriously ill for some time after the operation. If the freezing-point is $-0{\cdot}60°$ C. nephrectomy is inadmissible.

**Movable Kidney.**—C. A. McWilliams [2] has contributed a paper on the results of operation for this condition in the Presbyterian Hospital in New York. The article is based on 61 cases of operation. As to the frequency of movable kidney the statistics of Glenard are quoted. That writer, in an examination of 4,215 patients, found 14 per cent. of all patients, male and female, to be affected, and 22 per cent. of all the females. The degree of mobility is least where half the kidney is palpable, and most marked where the organ is found in the iliac fossa or the pelvis. Only two of the 61 cases operated upon were in males. The histories showed that excessive child-bearing had not been a factor in causing the mobility of the kidney. The right kidney was affected in 86·8 per cent. of the 61 cases, the left in 6·6 per cent., and both kidneys in 6·6 per cent. In seven cases a history of trauma antedated the symptoms, and in two cases there was marked emaciation, but in the remainder no cause was discovered. In nine cases it was noted that pressure on the superior mesenteric vein had brought about congestion in the cæcum. Gastro-intestinal disturbances were frequently observed. Urinary symptoms were not prominent in the cases, but nine cases of them showed increased frequency of micturition, four had dysuria, and two suffered from occasional hæmaturia. Many showed symptoms of reflex neuroses, such as nervousness, fatigue, headache, depression, insomnia, and palpitation. In five cases there was general enteroptosis. The condition has to be carefully distinguished from (1) distended gall bladder; (2) new growths, including pyloric cancer; (3) retained fæces; (4) pancreatic cyst; (5) ovarian tumours; (6) renal tumours and calculi; and (7) "Riedel's" lobe of the liver. The author agrees with Israel, that operative treatment is only definitely indicated in those cases in which there are colicky attacks which are the result of pulling or kinking of the pedicle, particularly if there is retention of urine in the kidney. The Weir-Mitchell rest-cure will improve the nervous symptoms. An abdominal support *without* a kidney cushion is the only form to be used. Two of the 61 cases died as a result of the operation, one from peritonitis the other from pulmonary embolism. Twenty-two cases were cured, eight greatly benefited, seven slightly improved, and five not benefited. Rees Phillips [3] records a case of floating kidney in an infant. Such a condition is very rare. The left kidney glided about freely in the abdomen. The right kidney was movable but not floating. The mobility of the kidneys was discovered a few days after birth. The abdominal walls were lax and atonic. The infant was liable to attacks of greenish vomiting with collapse. A mesonephron must have been present on the left side.

**Intermitting Hydronephrosis.** — J. Michalski [4] has written an exhaustive paper on this subject. His conclusions are summarised as follows: (1) By intermittent hydronephrosis is understood a retention of urine in the pelvis of the kidney due to an obstruction which periodically subsides or which can be removed or surmounted by the pressure of the urine. (2) It may be produced by dislocation of the kidney and the changes which are thus brought about; by renal or ureteral calculi, by primary or secondary changes in the ureter from compression or displacement, or from traumatism. In several cases the cause could not be discovered. (3) The condition is characterised by periodic attacks of pain in the renal region accompanied by disturbances of general health, by swelling of the corresponding side of the abdomen and diminution of the quantity of urine passed, which symptoms more or less quickly disappear to return again after an interval. (4) The prognosis is serious, and treatment must be active, as spontaneous cure is extremely seldom observed. (5) Operative treatment to remove the cause is usually necessary. Nephrectomy is only indicated in certain special cases.

[1] Scot. Med. and Surg. Jour., Feb., 1903, p. 63. [2] Vide abst. Med. Chron., Dec., 1902, p. 208. [3] Lancet, March 14, 1903, p. 731. [4] Vide abst. Centralb. f. Chir., Jan 17, 1903, p. 79.

